# Comparative chemical and biological hydrolytic stability of homologous esters and isosteres

**DOI:** 10.1080/14756366.2022.2027933

**Published:** 2022-02-13

**Authors:** Hygor M. R. de Souza, Jéssica S. Guedes, Rosana H. C. N. Freitas, Luis G. V. Gelves, Harold H. Fokoue, Carlos Mauricio R. Sant’Anna, Eliezer J. Barreiro, Lidia M. Lima

**Affiliations:** a Instituto Nacional de Ciência e Tecnologia de Fármacos e Medicamentos (INCT-INOFAR), Laboratório de Avaliação e Síntese de Substâncias Bioativas (LASSBio®), Universidade Federal do Rio de Janeiro (UFRJ), CCS, Cidade Universitária, Rio de Janeiro, Brasil; b Pós-graduação em Química, Instituto de Química, Universidade Federal do Rio de Janeiro, Rio de Janeiro, Brasil; c Instituto de Tecnologia em Fármacos – Farmanguinhos/FIOCRUZ, Rio de Janeiro, Brasil; d Departamento de Química, Instituto de Ciências Exatas, Universidade Federal Rural do Rio de Janeiro (UFRRJ), Seropédica, Brasil

**Keywords:** Esters, plasma and microsomal stability, homologous and isosteres

## Abstract

Esters are one of the major functional groups present in the structures of prodrugs and bioactive compounds. Their presence is often associated with hydrolytic lability. In this paper, we describe a comparative chemical and biological stability of homologous esters and isosteres in base media as well as in rat plasma and rat liver microsomes. Our results provided evidence for the hydrolytic structure lability relationship and demonstrated that the hydrolytic stability in plasma and liver microsome might depend on carboxylesterase activity. Molecular modelling studies were performed in order to understand the experimental data. Taken together, the data could be useful to design bioactive compounds or prodrugs based on the correct choice of the ester subunit, addressing compounds with higher or lower metabolic lability.

## Introduction

Esters are one of the most explored functional groups in the preparation of prodrugs. Several bioactive compounds and drugs containing carboxylic acids and free hydroxyl groups exhibit limited oral bioavailability due to absorption and/or first-pass metabolism problems^1^^,^^2^. Ester pro-drugs usually display better tissue mobility and they are non-specifically hydrolysed by human esterases, such as carboxylesterases 1 and 2 (CES 1 and CES 2) to yield high circulating concentrations of the active component post absorption^3^^,^^4^. Therefore, the presence of an ester group in the structure of bioactive compounds is often associated with metabolic instability. In fact, ester groups could be introduced during the design of soft drugs, defined as therapeutically active compounds that undergo a predicted fast metabolism into inactive metabolites after exerting their desired therapeutic effects^5^.

Given the role of ester groups in the design of pro-drugs or soft drugs, several authors have focussed on understanding the possibility to modulate ester stability by modifying the nature of alkoxyl substituent (RCOOR1) to yield compounds with variable metabolic labilities^6–11^.

**Figure 1.**
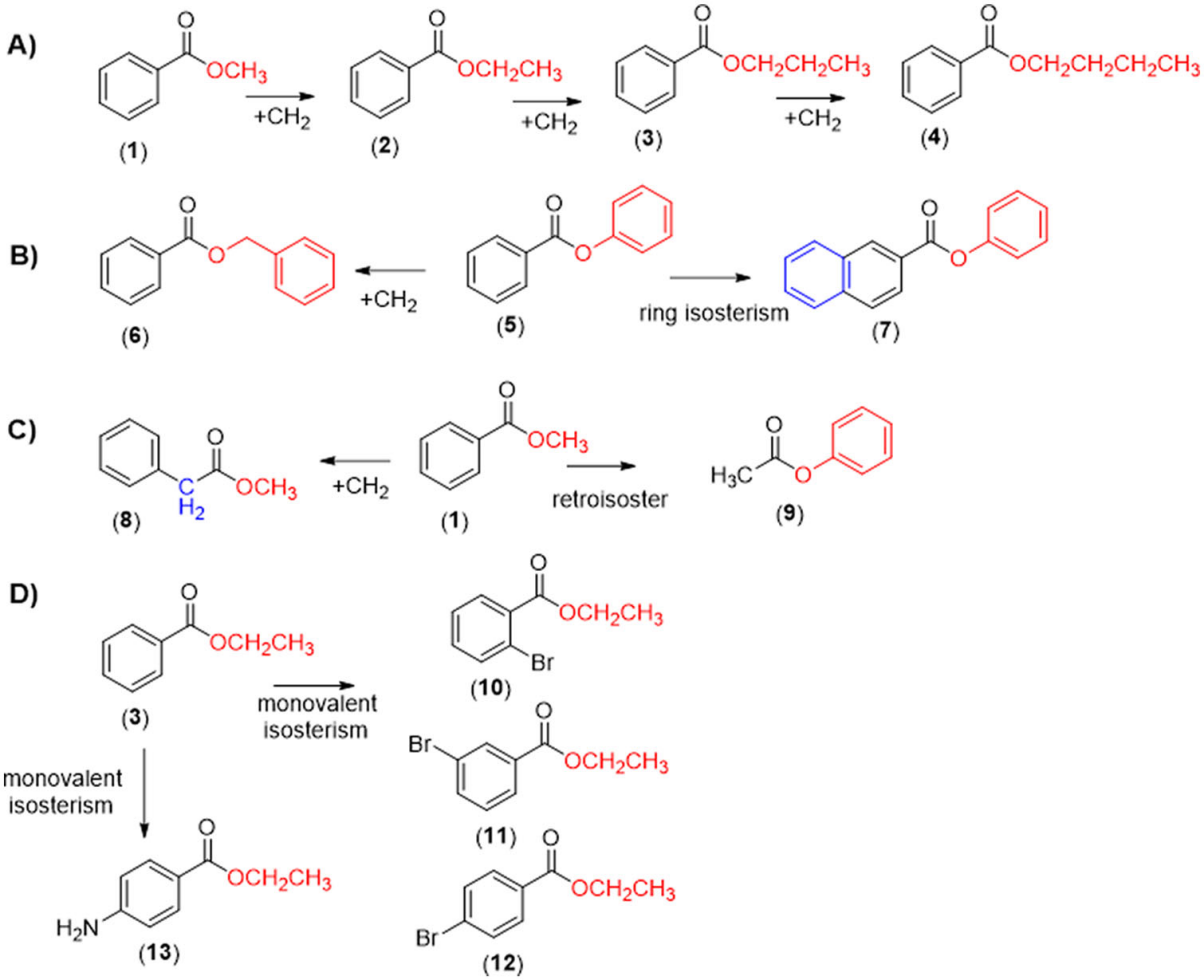
Design concept of the target benzoate esters **1**–**13** by applying linear homologation strategy (A); homologation and retroisosterism (B and C) and monovalent isosterism (D).

In the attempt to contribute to that discussion, we describe a comparative study of the chemical and biological hydrolysis of benzoate esters and analogues (**1**–**13**, Figure 1), aiming to identify the role of homologation and isosterism strategies^12–14^, in the lability of the target compounds and to establish an eventual structure hydrolytic lability relationship.

## Materials and methods

### Chemicals and reagents

The compounds methyl benzoate (1), *n*-propyl benzoate(3), *n*-butyl benzoate (4), methyl 2-naphthoate (**7**), phenylacetate (**9**), ethyl 2-bromobenzoate (**10**), ethyl 3-bromobenzoate (**11**), ethyl 4-bromobenzoate (**12**), methyl 8-fluoro-5-methyl-6-oxo-4*H*-imidazo[1,5-a][1,4]benzodiazepine-3-carboxylate (methyl flumazenil ester, **16**), methyl 4-aminobenzoate (methyl benzocaine ester, **14**), and methyl [1,1′-biphenyl]-4-carboxylate (**17**) were synthesised at the Laboratório de Avaliação e Síntese de Substâncias Bioativas (LASSBio®, UFRJ, Brazil) with degree of purity ≥98%. Flumazenil (**13**) was purchased from Cristalia® – Brazil. Formic Acid (96%), acetonitrile and methanol of HPLC grade were purchased from Tedia®-Brazil. Water used for the HPLC analysis was produced using a water purification system (Master System MS2000, Gehaka, Brazil). Ethylbenzoate (**2**), phenylbenzoate (**5**), benzylbenzoate(**6**), methyl 2-phenylacetate (**8**), ethyl 4-aminobenzoate (benzocaine, **13**), bicinchoninic acid kit for protein determination, ethylenediamine-tetra-acetic-acid (EDTA), monobasic and bibasic potassium phosphate, bis(*p*-nitrophenyl) phosphate sodium salt, magnesium chloride hexahydrate, β-nicotinamide adenine dinucleotide phosphate (NADP), D-glucose-6-phosphate, glucose-6-phosphate dehydrogenase, tetrahydrofuran (THF) and lithium hydroxide (LiOH) were purchased from Sigma Aldrich (St. Louis, MO, USA).

### Preparation of solutions and standards

The stocks solutions of test compounds and internal standard (IS) were prepared in dimethyl sulfoxide (DMSO) at a concentration of 100 mM and stored at −20 °C.

### Base-catalysed hydrolysis study

The study of chemical hydrolysis with the hydroxyl ion promotion was performed by adding 5 mg of each test compound (**1**–**12**) in a microtube containing tetrahydrofuran and water (THF: H2O, 6:4). The reaction was started by adding LiOH (24 μL, 1 mol/l), and the mixture was incubated at 37 °C for 5, 10, 20, and 30 min, under stirring in a water bath. At the end of the incubation period, an aliquot (20 μL) was transferred to a new tube, and subsequently, HCl (0.3 μL, 10% v:v) was added. The pH of the sample was checked to verify neutralisation, and acetonitrile (480 μL) was added. The final concentrations of all compounds in the solution were 2,5 g/L. Finally, the samples were filtered through PVDF syringe filters (Millex®: 0.22 µm pore size) and analysed by HPLC^15^.

### Rat plasma hydrolytic study

Rat plasma was obtained from male Wistar rats’ blood and diluted in phosphate buffer (pH 7.4) to obtain a final plasma concentration of 80%. After that, the test compounds (1–16) were added at a final concentration of 500 μM with 250 μL of the final volume. The sample was incubated at 37 °C for 5, 10, 20, and 30 min, under stirring. At the end of the incubation time, the reaction was stopped by adding methanol (500 μL) and acetonitrile (500 μL), both in ice-cold temperature. The mixture was centrifuged at 13.500 rpm for 15 min at 4 °C. The organic phase was transferred to a microtube, and the IS (1.25 µL) was added. Finally, the samples were filtered through PVDF syringe filters (Millex®: 0.22 µm pore size) and analysed by HPLC^16^.

### Hydrolytic study using rat liver microsomes

Rat liver microsomes were obtained from male Wistar rats (280–330 g) and prepared according to the methodology described by Cabrera and co-workers^17^. After obtaining rat liver microsomes, the protein quantification was done by a bicinchoninic acid assay using a commercial protein determination kit obtained from Sigma-Aldrich (St Louis MO, USA)^18^. In a microtube, 1 mg/mL of rat microsomal fraction was added together with each test compound (**1**–**16**) (1,25 µL, stocks solutions) and PBS in enough volume to complete 248.75 µL. Then, the samples were incubated at 37 °C for 5, 10, 20, and 30 min, under stirring. The reaction was stopped by adding organic solvents at ice-cold temperature (i.e., 500 µL of methanol and 500 µL of acetonitrile). Samples were centrifuged at 13.500 rpm for 15 min at 4 °C, the organic phase was collected, and the IS (1.25 µL) was added. In the end, the samples were filtered through PVDF syringe filters (Millex®: 0.22 µm pore size) and analysed by HPLC.

### Enzymatic inhibition study

The enzymatic pathway involved in the metabolic hydrolysis of the target esters (**1**–**16**) was identified by *in vitro* experiments, using rat plasma and liver microsomes, in the presence of irreversible selective inhibitor of carboxylesterase (CES) (bis-(*p*-nitrophenyl)phosphate) at 1, 10 and 100 µM^19^^,^^20^.

The inhibitor was pre-incubated for 30 min at 37 °C, with (*i*) plasma (*ii*) rat liver microsomes and phosphate buffer pH 7,4 (final volume of 250 µL). Then, the ester compounds (**1**–**16**) were added to the system at a final concentration of 500 µM. The mixture was incubated again at 37 °C for 90 min under agitation. The analysis was performed by the same HPLC method used in the previous experiments. The effect of CES inhibitor on the hydrolytic profile of each ester compound (**1**–**16**) was compared to a positive control (absence of CES selective inhibitor). Data were expressed as percentages of inhibition.

### Ethical considerations

All experimental procedures were approved by the Institutional Ethics Committee on the use of animals in scientific experiments at the Federal University of Rio de Janeiro (Protocol number: 028/15).

### HPLC method for analysis of ester concentration

A Shimadzu Prominence HPLC system (Shimadzu, Tokyo, Japan) consisting of a UV/VIS Photodiode Array Detector (SPD-M20A), a binary pump (LC-20AD), a vacuum degasser (DGU-20A5), and an autosampler (SIL-20A) was used for analyses. The Chromatographic separation was achieved using a reversed phase (Kromasil® C18 HPLC Column: particle size 5 µm, L × I.D. 250 × 4.6 mm) preceded by a guard column (Phenomenex® C18: 4 × 3 mm). The column was held at ambient temperature (22 ± 2 °C). For the selection of the wavelength, the diode array-detector was used. To avoid interference with impurities, the longest suitable wavelength was chosen to evaluate the chromatograms, which allowed for sensitivity. The mobile phase was a combination of eluent A (0.1% formic acid) and eluent B (acetonitrile). Three mobile phases were carried out using an injection volume of 20 µL and an flow rate set at 1.0 ml/min. In this work, the mobile phases used were: (*i*) Compounds **1**–**7** and **10**–**12**: 0–12 min linear gradient from 50% A to A:B 10:90 v/v; 12–18 min A:B 10:90 v/v. (*ii*) Compounds **8**–**9**: 0–15 min. A:B 40:60 v/v. (*iii*) Compounds **13**–**16**: 0–4 min. A:B 90:10 v/v; 4–8 min. linear-gradient from 90% A to A:B 60:40 v/v; 8–12 min. A:B 60:40 v/v.

All analytical methods were validated in accordance with guidelines of the Brazilian Regulatory Agency, ANVISA^21^ and Food and Drug Administration, FDA^22^. The analytical parameters evaluated were specificity, linearity, sensitivity, recovery, intra-day and inter-day precision and accuracy (See Supplemental data).

### Molecular modelling studies

All compounds were constructed with Spartan’16 (Wavefunction, Inc.). A Monte Carlo conformational search was performed with the molecular mechanics' method MMFF (Merck Molecular Force Field). The geometry of the lowest energy conformer of each compound was re-optimised with the semi-empirical method PM6^23^. The LUMO energy, atomic coefficients, and the Mulliken charges were calculated from the lowest energy conformer. The molar refractivity of the compounds (**1**–**12**) was determined using the Lorentz-Lorens relationship^24^^,^^25^.

Due to the impossibility of carrying out the molecular modelling studies with the rat CES, since there is no crystallographic structure in the Protein Data Bank (PDB), we chose a CES from another mammalian species, *Homo sapiens*, 1YAJ (resolution: 3.2 Å)^26^. The identity amongst human liver carboxylesterase 1 (hCE1) and rat carboxylesterase is 77.6% for the primary sequence and 75.0% for amino acids of the binding site, when calculated with uniprot server (https://www.uniprot.org/align/). The primary and binding site sequence alignment of human and rat carboxylesterase is showed in the supplementary material (Figure S1 and S2). The GOLD 5.6 program (CCDC) was used for the docking runs, and the four fitness score functions ASP, ChemPLP, ChemScore, GoldScore, were evaluated by redocking the co-crystallized ligand benzoic acid^27–30^. The crystallographic water molecules were removed during the docking runs, and the binding site was determined within 6 Å around the benzoic acid ligand. The covalent docking mode was used for the substrate molecules with the protein link at the catalytic Ser221 hydroxyl group and the substrate link atom at the carbon of ester groups. Docking runs were performed in triplicate and the poses presenting the best scores were analysed to identify potential interactions with amino acid residues located at the enzyme’s binding site.

**Figure 2.**
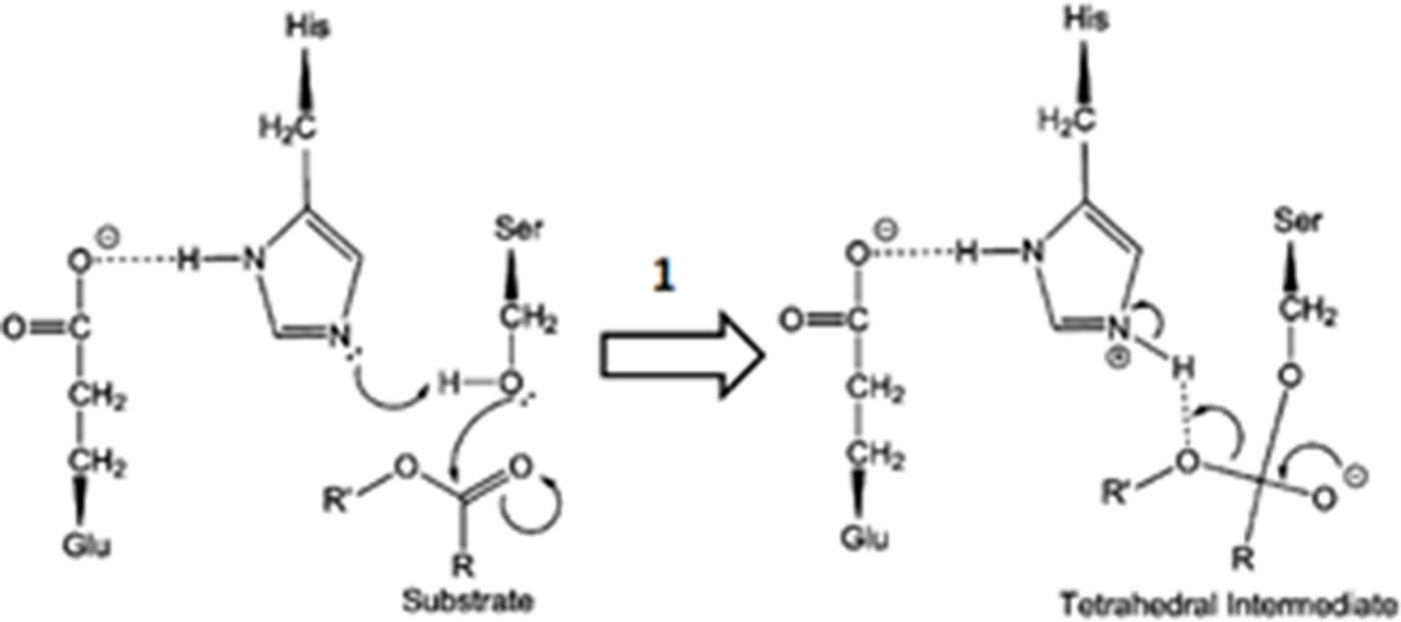
Proposed first step of the catalytic mechanism of hydrolysis catalysed by CEs (adapted from Yu and collaborators^42^).

Regarding the possible hydrolysis mechanism of benzoate esters by hCE1, we select step 1 (Figure 2) that represents the formation of the first tetrahedral intermediate, involving the catalytic triad, as the rate-determining step^31^^,^^32^. The following procedure evaluated the activation energy (E_a_) for this step: initially, the geometries of the best poses of the tetrahedral intermediates (Figure 2) obtained by covalent docking, were reoptimized with the semiempirical molecular orbital approach with the PM6-DH2 Hamiltonian available in MOPAC2016 (Stewart Computational Chemistry). The semiempirical molecular orbital method enables fast quantum mechanical calculations, and the choice of the PM6-DH2 for the E_a_ calculation was based on the fact that it is a modification of the original PM6 Hamiltonian containing the first-generation correction for dispersion interactions in the form of a pairwise interatomic force field and a second-generation H-bonding empirical correction. The performance of the corrections with several test sets for noncovalent interactions was shown to reach the quality of the DFT-D approaches^33^.

To reduce the computational cost, only amino acid residues with at least one atom located within 6 Å from the ligand were selected for geometry optimisation. As for the base-catalysed reaction, the calculations were performed with the solvent continuum model using the water dielectric constant, 78.4^23^^,^^34^. After the optimised geometry of the complex containing the tetrahedral intermediate was obtained, we performed a reaction path calculation by stretching the bond between the Ser221 O-atom and the tetrahedral intermediate C-atom with an increment of 0.1 Å, with a total of 40 points, until the unliganded substrate-enzyme complex geometry was obtained, determining the corresponding heat of formation (ΔH_f_) for each point. Then, the two geometries adjacent to the point with the highest ΔHf in the reaction path were used as initial guesses in a calculation with the LOCATE-TS routine^35^ for finding rough transition state (TS) geometries. The final TS geometry refinement was carried out in cartesian coordinate space with the TS routine^36^.

A true transition state is a stationary point on the reaction path that has precisely one negative force constant and the related vibrational frequency is imaginary. So, after the geometry optimisation, the vibrational frequencies of the atoms in the region of the reaction site were calculated using a FORCETS calculation^35^. If one of these frequencies is large and imaginary (printed as a negative number), and all the rest are positive or small and imaginary, then the system is confirmed to be a transition state^35^^,^^37^. With the ΔH_f_ values calculated for the optimised geometries of the unliganded substrate-enzyme complexes and the transition states, the E_a_ for each reaction was finally calculated.

To study the alkaline hydrolysis of the benzoate esters, we select the addition of a hydroxide ion to the carbonyl group leading to the tetrahedral intermediate as the rate-determining step, assuming the acyl-oxygen cleavage (B_AC_2) mechanism, according to Ingold proposed classification of ester hydrolysis mechanisms^38^^,^^39^. A procedure like that adopted for the enzymatically catalysed reaction was used to evaluate the corresponding relative activation energy (E_a_) for the tetrahedral intermediate formation step, but in this case, only a hydroxide ion and each benzoate ester molecules participated in the process. All the calculations were performed with the solvent continuum model using the water dielectric constant, 78.4^23^^,^^34^.

### Structural analysis of hydrolysis products

LC/MS was performed in the Shimadzu Prominence HPLC system (Shimadzu, Tokyo, Japan) described above coupled to electrospray ionisation mass spectrometry (HPLC-ESI-MS) model Esquire 6000-ESI Ion Trap MSn System Bruker Daltonics. The ESI source parameter settings were mass range m/z 100–500, capillary voltage 3500 V, nitrogen gas flow 6.0 L/min, temperature 250 °C, and nebuliser 4.0 psi.

### Statistical analysis

Values are expressed as mean ± standard error and analysed using GraphPrism software (version 5.0). One-way analysis of variance (ANOVA) followed by Dunnett post-tests was used for comparisons among groups. *P*-values ≤ 0.05 was considered as significant for all assays.

## Results and discussion

### Alkaline hydrolysis

Compounds **1**–**12** were submitted to hydrolysis by treating with lithium hydroxide, using a mixture of THF and H_2_O, and incubation at 37 °C under stirring. The time in minutes required to reduce half the initial amount of each ester compound (*i.e.,* half-life - t_1/2_) was calculated using the expression t_1/2_ = 0.693/a, with "a" being the slope of the natural log of concentration of the sample *vs*. the incubation time.

**Figure 3.**
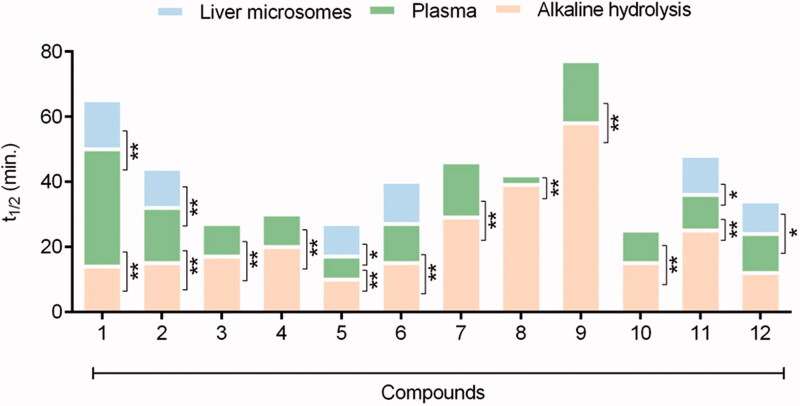
Comparative profile of compounds 1–12 in non-enzymatic hydrolysis using LiOH/THF:H2O, 37 °C and enzymatic hydrolysis using rat plasma and liver microsomes. Data correspond to mean ± SD (*n* = 3). **p-values ≤ 0.05 and *0.05 < *p*-values < 0.1.

As expected, when compared to the linear homolog’s esters **1**–**4**, the phenyl benzoate 5 displayed the lower hydrolytic stability (Figure 3(A)). The rapid hydrolysis of the phenyl benzoate (**5**, t_1/2_ = 11 min) can be explained by the lower energy of its LUMO orbital (Table 1) and the stability of the phenoxide ion stabilised by resonance. The kinetic of base hydrolysis of esters **1** (t_1/2_ = 14 min), **2** (t_1/2_ = 14 min), **3** (t_1/2_ = 19 min), and **4** (t_1/2_ = 21 min) seems to be directly related to the size of the alkyl group, as can be seen by their comparative molar refractivity (Table 1). The inductive electron donor effects of the alkyl group increase the strength of the alkoxide ion and decrease its stability. The comparison between the kinetics hydrolysis of **1** (t_1/2_ = 14 min) versus its benzyl analog **6** (t_1/2_ = 16 min) and versus the phenyl benzoate **5** (t_1/2_ = 11 min) corroborates the hypothesis that the rate-determining step during the base-catalysed hydrolysis is the elimination of the alkoxyl group^40^^,^^41^. These data also suggest a low influence of steric effects on the base hydrolysis process since the ester **6** that has the highest molar refractivity (Table 1). Therefore, a higher steric effect, displayed a half-life lower than esters **3** and **4**. The kinetics of hydrolysis of the esters substituted at the *ortho* (**10**), *meta* (**11**), and *para* (**12**) position by bromine atom, a classical electron-withdrawing substituent, has been established. As shown in Table 2, lower hydrolytic stability for ethyl *p*-bromo benzoate (**12**; t_1/2_ = 12 min) was observed when compared to its unsubstituted analogue **2** (t_1/2_ = 14 min). An opposite result was found for ethyl *m*-bromo benzoate (**11**, t_1/2_ = 25 min), in agreement with the higher energy of its LUMO orbital (Table 1), due to the electron donor effect exerted by the bromine when connected to the meta position of the aromatic system, thus reducing carbonyl electrophilicity. Ethyl 2-bromo benzoate (**10**, t_1/2_ = 15 min) exhibited higher stability than the ethyl 4-bromo benzoate **12** (t_1/2_ = 12 min), and comparable hydrolytic stability to unsubstituted ethyl benzoate **2** (t_1/2_ = 15 min). This data may indicate competition between the electronic aspect and unfavourable steric events of the bromine atom in the *ortho* position of the carbonyl group. As shown in Figure 3 and Table 2, the base kinetic hydrolysis of esters **1**–**12** (established through their t_1/2_ values) correlates directly to the theoretical results calculated by *in silico* approaches and summarised in Table 1.

**Table 1.** Atomic coefficient values, energy levels of LUMO orbitals and Mülliken charge of carbonyl carbon atom, and molar refractivity of compounds **1**–**12** calculated by the SPARTAN 14’ program (Wavefunction Inc.)

Compounds	Atomic coefficient	LUMO (eV.)	Mülliken charge	Molar refractivity
1	0.1282	2.590	0.7500	37.80
2	0.1277	2.590	0.7540	42.59
3	0.1292	2.590	0.7580	47.24
4	0.1276	2.600	0.7530	51.88
5	0.1395	2.480	0.7230	57.89
6	0.1302	2.590	0.7580	61.55
7	0.0722	2.060	0.7680	57.75
8	0.0182	3.830	0.7160	42.08
9	0.0069	3.600	0.6670	37.45
10	0.1058	2.390	0.7070	50.53
11	0.0914	2.640	0.6700	50.30
12	0.1060	2.220	0.7580	50.53

**Table 2.** Comparative stability of esters **1**–**16**, expressed by their half-life values, determined in base, rat plasma and rat liver microsomes hydrolysis conditions. Abbreviation: N.D.: Not Determined.

Compounds	Hydrolysis t_1/2_ (min.)		
	Alkaline	Rat	Rat
		plasma	liver microsomes
methyl benzoate (1)	14	36	15
ethyl benzoate (2)	14	17	12
n-propyl benzoate (3)	19	10	N.D.
n-butyl benzoate (4)	21	10	N.D.
phenyl benzoate (5)	11	7	10
benzyl benzoate (6)	16	12	13
methyl 2-naphthoate (7)	29	17	N.D.
methyl 2-phenylacetate (8)	39	3	N.D.
phenylacetate (9)	58	19	N.D.
ethyl 2-bromobenzoate (10)	15	10	N.D.
ethyl 3-bromobenzoate (11)	25	11	12
ethyl 4-bromobenzoate (12)	12	12	10
Benzocaine (13)	N.D.	13	N.D.
methyl benzocaine ester (14)	N.D.	27	N.D.
flumazenil (15)	N.D.	89	N.D.
methyl flumazenil ester (16)	N.D.	238	N.D.

### Rat plasma and liver microsome hydrolysis

The biologic hydrolysis of the esters **1**–**12** was carried out using rat plasma and rat liver microsomes. Each compound's half-life (t_1/2_) was calculated using the expression t_1/2_ = 0.693/a, as previously described. The kinetics of biological hydrolysis can be visualised in Table 2 and in Figure 3 from the comparative half-life values.

The results indicate that among the linear homologous esters (**1**–**4**), methyl benzoate (**1**) has the higher plasma metabolic stability (t_1/2_ = 36 min). With the exception of methyl- (**1**, t_1/2_ = 36 min) and ethyl benzoate (**2**, t_1/2_ = 17 min), all the remaining esters (**3**–**12**) showed higher hydrolytic lability in rats’ plasma than under base hydrolysis conditions (Table 2). Plasma stability was inversely proportional to the size of the alkoxyl group (OR): methyl- (**1**, t_1/2_ = 36 min), ethyl- (**2**, t_1/2_ = 17 min), n-propyl- (**3**, t_1/2_ = 10 min), n-butyl (**4**, t_1/2_ = 10 min) and phenyl (**5**, t_1/2_ = 7 min) (Table 2).

Interestingly, the homologation strategy that resulted in the conjugation breaking between the carbonyl and the phenyl group of the methyl benzoate (**1**) and in the greater conformational freedom produced a significant loss of metabolic stability, being the homolog **8** twelve times less stable than ester **1** (Table 2 and Figure 3).

Electronic factors, resultant from the substitution of the phenyl ring of ethyl benzoate (*e.g.,* **10**–**12**), seem not to interfere in the hydrolysis catalysed by plasma enzymes, differently from the result found from chemical hydrolysis (Table 2). Compounds **10**–**12**, having the substitution of the bromine atom in the ortho, meta, and para position of the phenyl ring, respectively, showed a very similar plasma metabolisation rate (Table 2 and Figure 3). This finding was corroborated by comparing the rate of plasma hydrolysis of compound **12** (showing a withdrawing electron group) versus the benzocaine **13** (containing a donor electron group). Compounds **12** and **13** had a plasma half-life of 12 min and 13 min, respectively (Table 2).

Our data suggest that the presence of aromatic rings with higher electron density decreases plasma metabolic lability. The phenyl-2-naphthoate (**7**, t_1/2_ = 17 min) showed greater stability to plasma hydrolysis than its isostere phenyl benzoate (**5**, t_1/2_ = 7 min). On the other hand, the retroisosterism strategy seems to contribute unfavourably to the plasma stability of compound **9** (t_1/2_ = 19 min) when compared to ester **1** (t_1/2_ = 36 min) (Table 2).

Given those results, we decided to study the comparative metabolic behaviour of the esters **1** and **2**, **5** and **6**, **11** and **12**, using another biological matrix rich in carboxylesterases. Therefore, the kinetics of hydrolysis was established using rat liver microsomes in the absence of cofactor in order to prevent eventual oxidative metabolic reactions catalysed by CYP450 or FMO. As depicted in Table 2 and Figure 3, the methyl benzoate (**1**, t_1/2_ = 15 min) showed higher stability than the ethyl benzoate (**2**, t_1/2_ = 12 min). Compound **5** (t_1/2_ = 10 min) displayed a lower stability than its homologous **6** (t_1/2_ = 13 min), while the esters **11** (t_1/2_ = 12 min) and **12** (t_1/2_ = 10 min) exhibited similar hydrolytic lability. These results are qualitatively comparable to those found using rat plasma.

**Figure 4.**
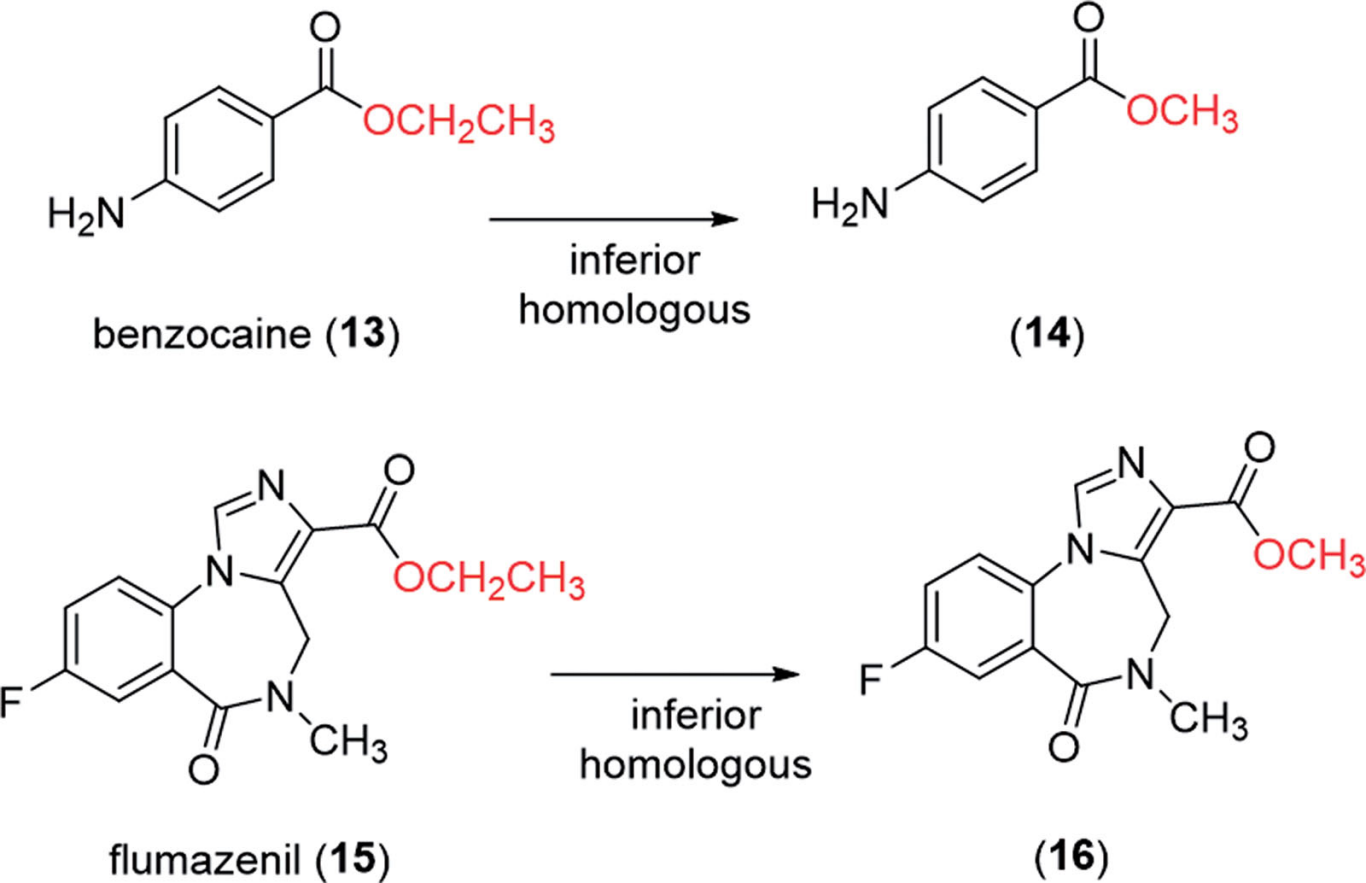
Chemical structures of benzocaine (**13**), flumazenil (**14**) and their inferior homologues **14** and **16**.

**Figure 5.**
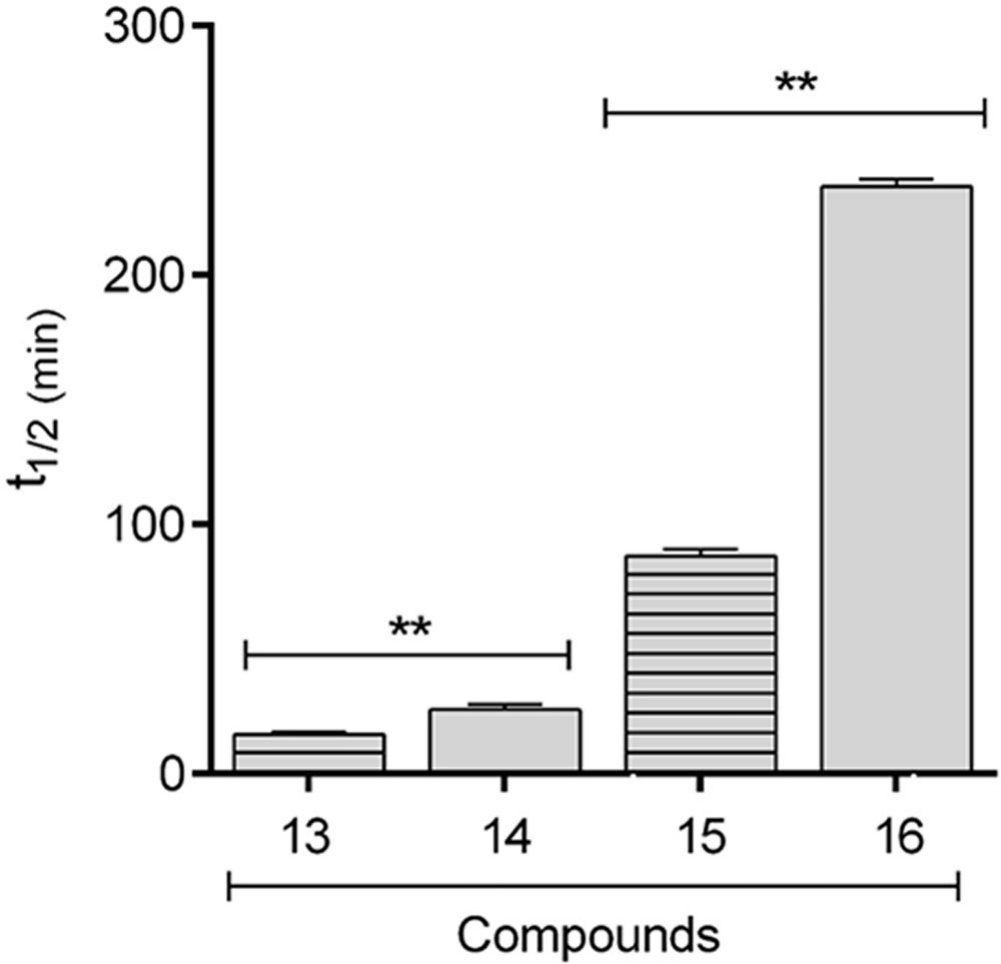
Comparative profile of compounds **13**–**16** in enzymatic hydrolysis using rat plasma. Data correspond to mean ± SD (*n* = 3). **p-values ≤ 0.05.

In order to verify if the greater stability of methyl ester, observed in the studied models, would be extrapolated to known drugs, the comparative hydrolysis kinetics of benzocaine (**13**) and flumazenil (**15**) and their inferior homologs **14** and **16** (Figure 4) was performed using rat plasma (Figure 5). The results confirmed the reactivity pattern proposed from the studies with the esters model **1** and **2**. Flumazenil (**15**) exhibited a plasma half-life of 89 min, being 2.7 times less stable to plasma hydrolysis than its methyl homologous **16** (t_1/2_ = 238 min). Similar results were found for benzocaine (t_1/2_ = 13 min) and its methyl homologous **14** (t_1/2_ = 27 min) (Table 2, Figure 5).

### Enzymatic inhibition study in rat plasma and rat liver microsomes

**Figure 6.**
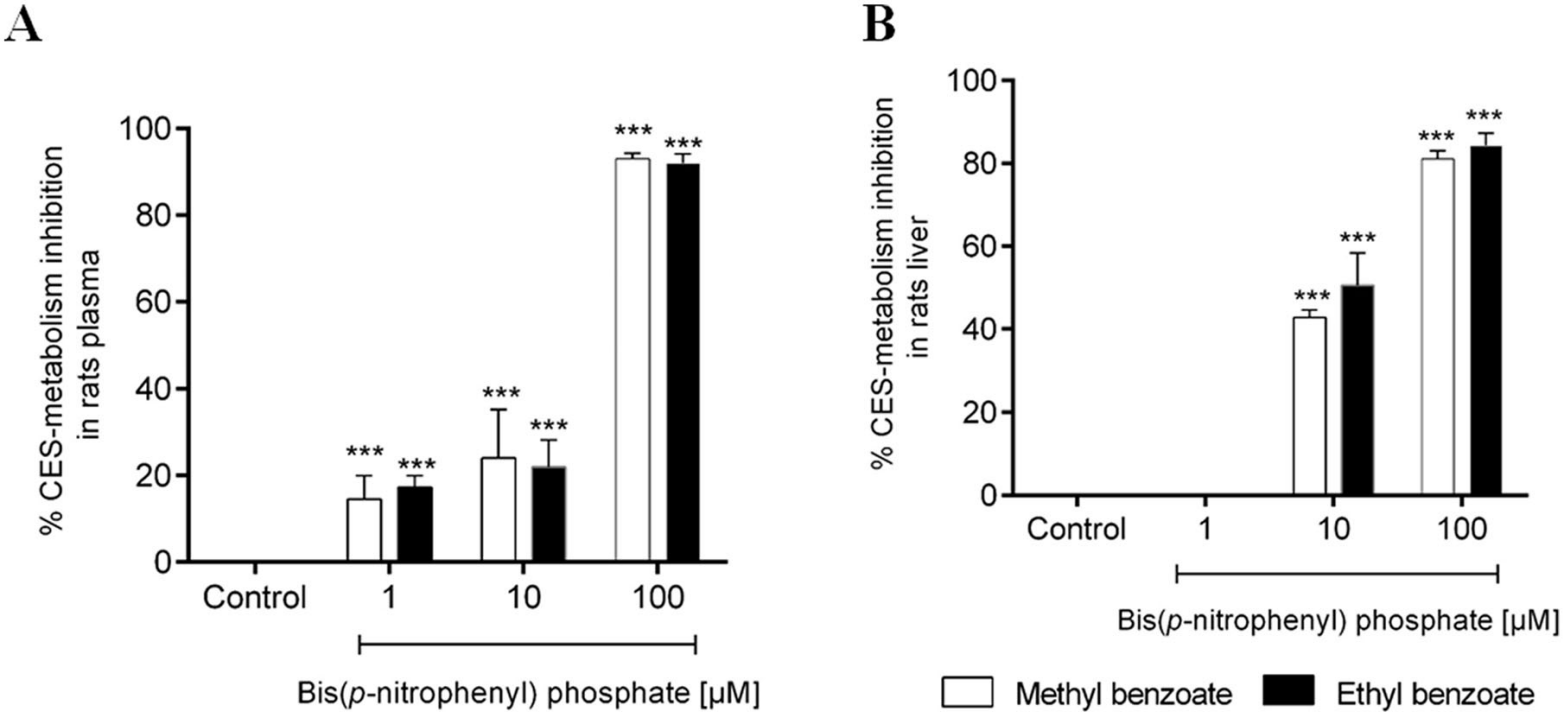
Percentage inhibition of *in vitro* plasma (A) and microsomal hepatic metabolism (B) of methyl benzoate and ethyl benzoate by selective inhibitors of carboxylesterase enzymes (Bis(*p*-nitrophenyl) phosphate at 1, 10, 100 μM), compared with the control group (absence of inhibitor). The data represent the mean of triplicate incubations. Values are mean ± SD. ***p-values < 0.001 compared with the control group analysed by ANOVA following a Dunnett post-test.

To confirm the participation of carboxylesterases (CES) in the metabolic stability of the target esters, the rat plasma hydrolysis experiment with compound **1** and **2** were carried out from the co-incubation with an irreversible and selective inhibitor of CES, the bis(*p*-nitrophenyl)phosphate. In the presence of the CES inhibitor, the metabolism of methyl benzoate (**1**) and ethyl benzoate (**2**) was strongly reduced in a concentration-dependent manner (Figure 6(A)). Similar results were found in the experiment using rat liver microsomes in the presence of the bis(*p*-nitrophenyl)phosphate (Figure 6(B)). These data confirmed the participation of CES in the hydrolytic metabolism of compounds **1** and **2** and can be extrapolated to the other esters studied in this paper.

### *In silico* studies

To understand the differences among the kinetic of base versus plasma hydrolysis, theoretical studies were carried out. For this purpose, the linear homologous esters **1–4** were selected.

**Figure 7.**
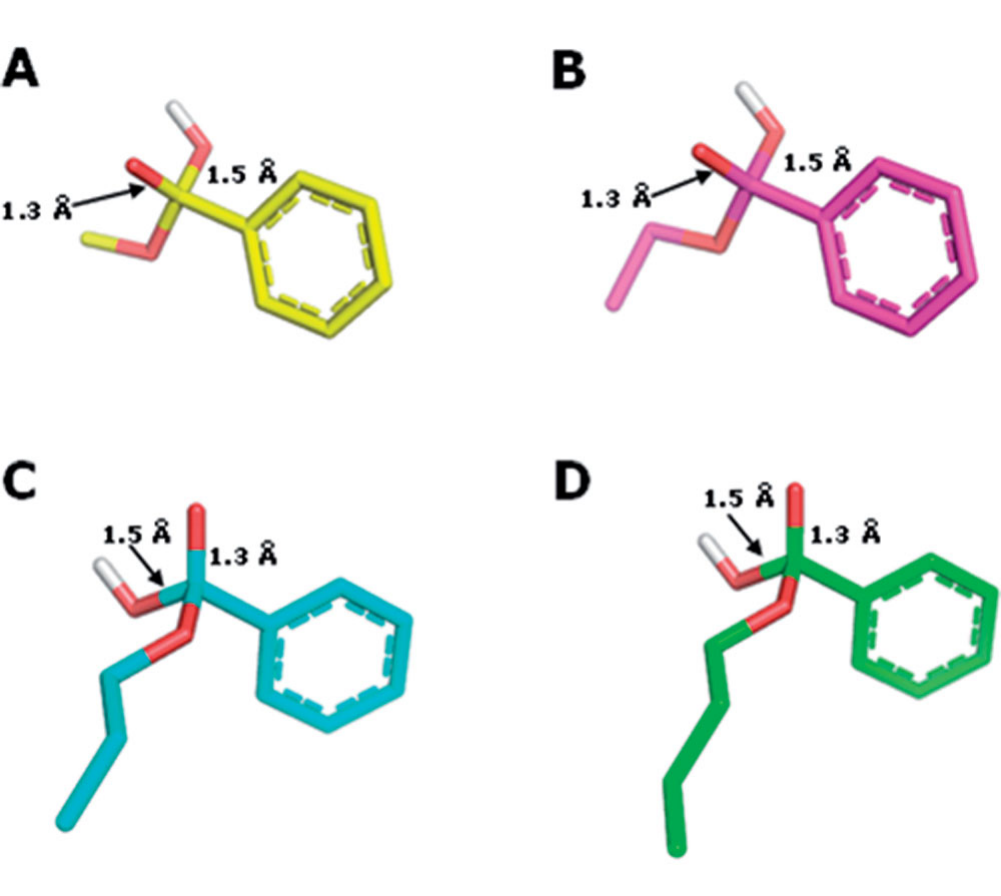
Structures of the tetrahedral intermediates generated from the alkaline hydrolysis for methyl (**1**, C atoms in yellow), ethyl (**2**, C atoms in magenta) n-propyl (**3**, C atoms in cyan) and n-butyl (**4**, C atoms in green) benzoates. O atoms in red and H atoms in white.

The enthalpy data for the reactant complexes and the transition states (TS), generated during the base catalysed hydrolysis, were calculated by the semi-empirical PM6-DH2 and were used to compute the activation energy of the base hydrolysis (Table 3 and Figure 7), according to the described methodology. The analysis of the kinetics of the tetrahedral intermediate formation, which is proposed as the rate-determining step of the base-catalysed hydrolysis^39^, was analysed. According to the Arrhenius kinetic theory, it is expected that a chemical reaction with a higher relative activation energy (E_a_) value would present a lower reaction rate constant (k) value and, consequently, a higher t_1/2_. The relative E_a_ values calculated with the PM6-DH2 method show that the necessary energy for the reactant complex to reach the TS in the base-catalysed hydrolysis is higher for the *n*-butyl- (**4**), followed by the n-propyl- (**3**), ethyl- (**2**) and methyl- (**1**) benzoates (Table 3). These data are in agreement with the experimental t_1/2_ values determined for compounds **1–4** (Table 2). The longer the alkyl group, the greater the electron density donation towards the carbonyl carbon atom. So, since in the first step of the acyl-oxygen cleavage (B_AC_2) mechanism a nucleophilic attack of the hydroxide anion on the carbonyl-carbon occurs (with the partial formation of a covalent bond between the carbonyl C atom and the hydroxide O atom formation in the corresponding TS), it is expected that the longer the alkyl group is, the more energetic the TS should be^12^.

**Table 3.** Activation energies (kJ/mol) for the formation of the tetrahedral intermediated for base hydrolysis calculated with PM6-DH2 with the water dielectric constant.

	Methyl benzoate (**1**)	Ethyl benzoate (**2**)	n-Propyl benzoate (**3**)	n-Butyl benzoate (**4**)
**ΔH_I_** (Reactant complex)	−923.99	−951.61	−974.16	−992.82
**ΔH_f_** (TS)	−923.91	−948.97	−964.45	−979.31
**ΔH^#^**	0.08	2.64	9.71	13.51
E_a_*	2.66	5.21	12.28	16.09

*E_a_ =ΔH^#^ + RT, T = 310.15 K; ΔH^#:^ Transiton state enthalpy of reaction. TS: transition state. T: Temperature. R: gas constant.

**Figure 8.**
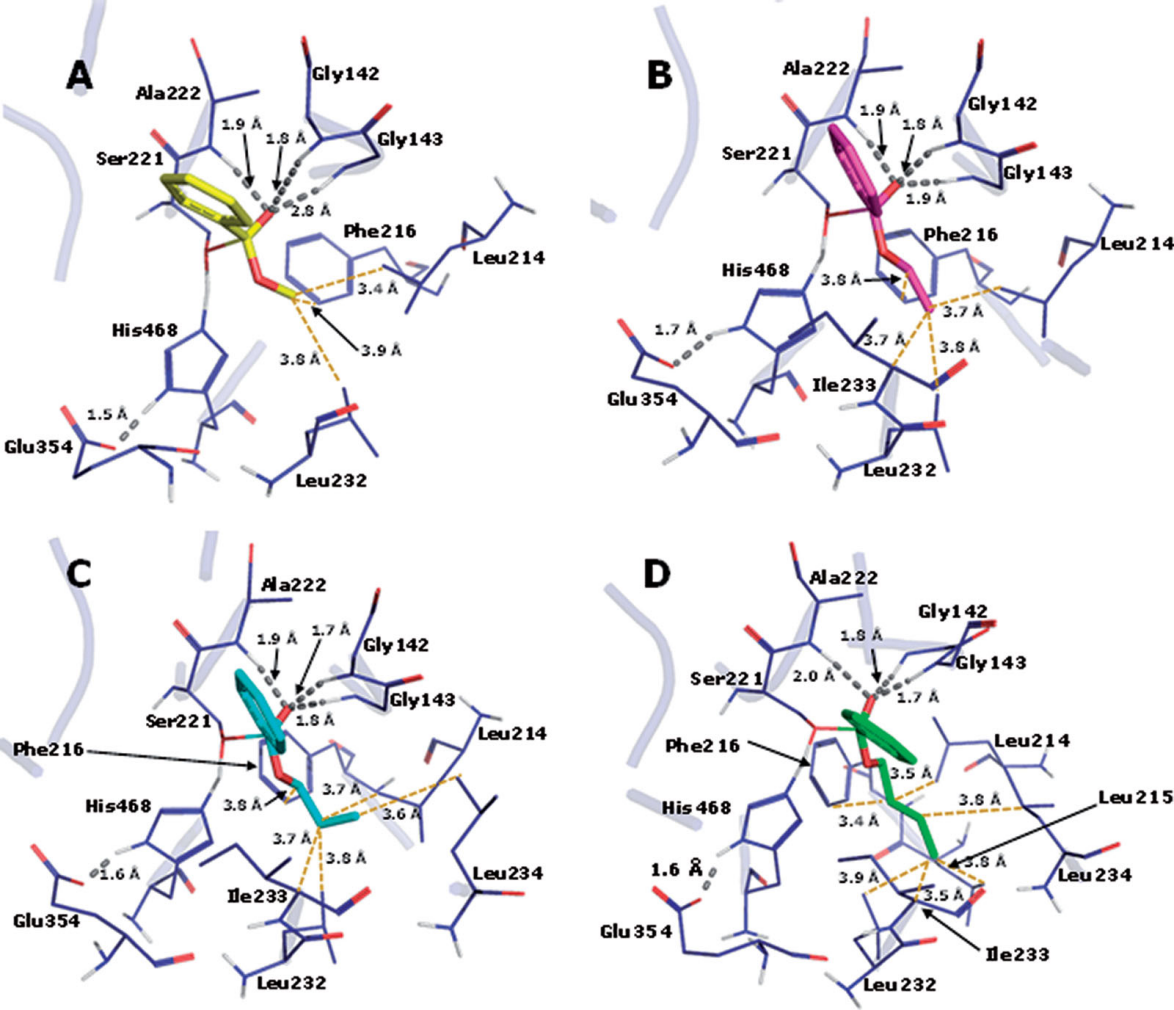
TS interaction profile in the binding site of hCE1 crystallographic structure 1YAJ, for methyl (**1**, C atoms in yellow), ethyl (**2**, C atoms in magenta) *n*-propyl (**3**, C atoms in cyan) and *n*-butyl (**4**, C atoms in green) benzoates. Hydrogen bonds are indicated in grey. O atoms in red and H atoms in white.

For the enzymatic hydrolysis study, we first defined the best docking conditions. The co-crystallized ligand (benzoic acid) was re-docked in the hCES. The best RMSD value, obtained with ChemPLP followed by rescoring with ChemScore, was 2.19 Å, which is better than 1YAJ crystallographic resolution (3.2 Å). This combination of functions was used for the docking runs, and the best resulting poses were used as input files for the semiempirical studies. After the semiempirical studies, the enthalpy data for the reactant complexes and the transition states (TS) were used to calculate the E_a_, as depicted in Table 4. Figure 8 shows the corresponding TS interaction arising from the reaction between esters **1–4** and amino acid residues from the catalytic site of hCE1 crystallographic structure.

**Table 4.** Activation energies (kJ/mol) for the formation of the tetrahedral intermediated for enzymatic hydrolysis calculated with PM6-DH2 with the water dielectric constant.

	Methyl benzoate	Ethyl benzoate	n-Propyl benzoate	n-Butyl benzoate
**ΔH_I_** (Reactant complex)	−9276.47	−9288.35	−9318.31	−9351.32
**ΔH_f_** (TS)	−9105.01	−9159.15	−9200.45	−9234.80
**ΔH^#^**	171.46	129.20	117.86	116.52
E_a_*	174.04	131.78	120.44	119.10
Dispersion energy in TS	−180.81	−183.74	−187.74	−189.80

*E_a_ =ΔH^#^+RT, T = 310.15 K; ΔH^#^: Transiton state enthalpy of reaction. TS: transition state. T: Temperature. R: gas constant.

The relative E_a_ values calculated with the PM6-DH2 method (Table 4) show that the energy necessary for the reactant complex to reach the TS in the CES-catalyzed hydrolysis is higher for the methyl- (**1**), followed by the ethyl- (**2**), *n*-propyl- (**3**) and *n*-butyl- (**4**) benzoates; being in accordance with the experimental t_1/2_ values determined in rat plasma.

In the enzyme-catalyzed reaction, the longer the alkyl group, the more stable the TS is. This suggests that the binding site should contain structural features that take advantage of the alkyl group volume to produce energetic stabilization. In fact, there is in the binding site a hydrophobic pocket composed of residues Leu214, Leu215, Phe216, Leu232, Ile233, and Leu234, where the alkyl group of each TS is inserted in (Figure 8). The pocket is progressively more occupied by the longer alkyl chain present in each ester substrate, which should increase dispersion forces helping stabilize the TS. In fact, as can be seen in Table 4, the dispersion energy present in the complexes between the enzyme and the TS becomes progressively more favorable as the alkyl chain of the esters increases.

Besides that, it can be seen in Figure 7 that the oxyanion hole, formed by the peptide NH groups of residues Gly142, Gly143, and Ala222, establishes H-bonds with the substrate carbonyl O atom that develops the negative charge as a result of the nucleophilic attack of the Ser221 alkoxide, which is also a stabilizing factor for the TS. It is noteworthy that the H-bond donated by Gly143 is getting progressively stronger in the transition states in the order methyl, ethyl, propyl, and butyl esters, as indicated by the length of the H-bonds (2.8, 1.9, 1.8, and 1.7 Å, respectively). This factor should also be related to the growing stability of the transition states in this series of ester substrates.

Taken together, our data indicate that in the linear homologous series the methyl ester (**1**) is the one with the lowest hydrolytic lability in rat plasma. The great metabolic stability of methyl esters when compared to their superior homologs was confirmed by the comparison between the kinetics of hydrolysis of the drugs flumazenil (**15**) and benzocaine (**13**), containing a carboxyethyl ester, and their lower homologs (**14** and **16**), comprising a carboxymethyl ester. The data also show that, except for the methyl ester (**1**) and the ethyl ester (**2**), the other homologs, isosteres, and retroisotere (**3–12**) displayed higher hydrolytic lability in rat plasma than under base-catalyzed hydrolysis.

The work provides evidence of the hydrolytic lability structure relationship in a biological medium rich in carboxylesterases (rat plasma and rat liver microsome), allowing that such information could be used in the design of bioactive compounds or prodrugs through the correct choice of ester subunit, aiming the desired modulation of the metabolic lability, targeting compounds with higher or lower plasma half-life.

## Supplementary Material

Supplemental Material
