# Supplementary material for: Comparative chemical and biological hydrolytic stability of homologous esters and isosteres
Source: J Enzyme Inhib Med Chem. 2022 Feb 13;37(1):718–27. doi: 10.1080/14756366.2022.2027933 (PMC8856110; doi:10.1080/14756366.2022.2027933)
Supplement: Supplemental Material [file IENZ_A_2027933_SM4593.pdf]

Supplemental Data

Comparative chemical and biological hydrolytic stability of homologous esters and isosteres

Hygor M. R. de Souza[a,b], Jéssica S. Guedes[b], Rosana H. C. N. Freitas[b], Luis G. V. Gelves[b], Harold H. Fokoue[c], Carlos Mauricio R. Sant´Anna[a,d], Eliezer J. Barreiro[a,b], and Lídia M. Lima[a,b]*

1

Method validation1

Specificity1

Linearity and sensitivity2

Accuracy and precision2

Recovery2

***Molecular modeling studies*..3**

Results and discussion..4

Table S1. Linearity and sensitivity data of esters compounds (**1-12**) 4

Table S2. Recovery, accuracy and precision data of esters compounds (**1-12**) **5**

Table S3. Selectivity data of esters compounds (**1-12**) **6**

Method validation

All analytical methods used in this study was validated in accordance with guidelines of the Brazilian Regulatory Agency, ANVISA (ANVISA, 2012) and Food and Drug Administration, FDA (US, 2013). The analytical parameters evaluated were: specificity, linearity, sensitivity, recovery, intra-day and inter-day precision and accuracy (See supplementary data).

Specificity

The specificity of the methods was determined by analysis of ten blank experiments (without esters compounds (**1**-**12**) and internal standard (IS)). After, blank samples spiked

with each ester (**1-12**) and its respective IS were evaluated by comparing chromatograms of blank samples.

Linearity and sensitivity

Linearity was assessed by standard curve in the range of 1.25-250 µM (LLOQ and 50-200% of ester assay concentration) and first point of its curve refers to lower limit of quantification (LLOQ 1.25-15 µM). The IS concentration was fixed (125 µM) in all analyses and each point was prepared in triplicate and analyzed in the same day. For construction of the calibration curves, peak area ratios of ester (**1-12**) and it's IS (prototype/IS) were plotted against analytic concentrations. The calibration curves were constructed using least-square linear regression and were obtained the data of determination coefficients (r^2), slope and intercept. It is important to emphasize that all linearity validation was prepared in the presence of the biological matrix (i.e. rat plasma or liver microsomes).

The sensitivity of this study was defined by the Lower Limit of Quantification (LLOQ) that was determined yielding a signal to noise ratio of at least 10 (S/N>10).

Accuracy and precision

Accuracy and precision were expressed as relative error (RE) and relative standard deviation (RSD), respectively. The intra-day precision and accuracy was verified in the presence of biological matrix by analysis of three replicates in three different concentrations (62.50, 125.00 e 156.25 µM) contemplating the linear interval of method. The inter-day precision and accuracy were determined in another day at the same concentrations. According to the guidelines (ANVISA, 2012; US, 2013) a method is considered precise and accurate with values of RSD ≤15% and RE ±15%.

Recovery

The recovery of each ester was determined by comparing the peak areas from extracted samples in the presence of biological matrix at three different concentrations (62.5, 125 and 156.25 µM) with those obtained by the replacement of biological matrix to organic solvent (acetonitrile and methanol, 1:1, v/v) with equivalent concentrations. Recovery values at 70-120% were considered appropriate.

Molecular modeling studies

The primary and binding site sequence alignment of human and rat carboxylesterase is showed below . (Figure S1 and S2). The alignement was performed by uniprot server (https://www.uniprot.org/align/).

```
CLUSTAL O(1.2.4) multiple sequence alignment
SP|P16303|EST1D_RAT  MRLYPLVWLFLAACTAWGYPSSPPVVNTVKGKVLGKYVNLEGFAQPVAVFLGIPFAKPPL 60
SP|P23141|EST1_HUMAN MWLRAFILATLSASAAWGHPSSPPVVDTVHGKVLGKFVSLEGFAQPVAIFLGIPFAKPPL 60
                     * *  ::   *:*.:***:*******:**:******:*.*********:***********
SP|P16303|EST1D_RAT  GSLRFAPPQPAEPWNFVKNTTSYPPMCSQDAVGGQVLSELFTNRKENIPLQFSEDCLYLN 120
SP|P23141|EST1_HUMAN GPLRFTPPQPAEPWSFVKNATSYPPMCTQDPKAGQLLSELFTNRKENIPLKLSEDCLYLN 120
                     * ***:********.****:*******:**  .**:**************::********
SP|P16303|EST1D_RAT  VYTPADLTKNSRLPVMVWIHGGGLVVGGASTYDGQVLSAHENVVVVTIQYRLGIWGFFST 180
SP|P23141|EST1_HUMAN IYTPADLTKKNRLPVMVWIHGGGLMVGAASTYDGLALAAHENVVVVTIQYRLGIWGFFST 180
                     :********:.*************:**.****** .*:**********************
SP|P16303|EST1D_RAT  GDEHSQGNWGHLDQVAALHWVQDNIANFGGNPGSVTIFGESAGGFSVSALVLSPLAKNLF 240
SP|P23141|EST1_HUMAN GDEHSRGNWGHLDQVAALRWVQDNIASFGGNPGSVTIFGESAGGESVSVLVLSPLAKNLF 240
                     *****:************:*******.***************** ***.***********
SP|P16303|EST1D_RAT  HRAISESGVVLTSALIT-TDSKPIANLIATLSGCKTTTSAVMVHCLRQKTEDELLETSLK 299
SP|P23141|EST1_HUMAN HRAISESGVALTSVLVKKGDVKPLAEQIAITAGCKTTTSAVMVHCLRQKTEEELLETTLK 300
                     *********.***.*:.  * **:*: **  :*******************:*****:**
SP|P16303|EST1D_RAT  LNLFKLDLLGNPKESYPFLPTVIDGVVLPKTPEEILAEKSFNTVPYIVGINKQEFGWIIP 359
SP|P23141|EST1_HUMAN MKFLSLDLQGDPRESQPLLGTVIDGMLLLKTPEELQAERNFHTVPYMVGINKQEFGWLIP 360
                     ::::.*** *:*:** *:* *****::* *****: **:.*:****:**********:**
SP|P16303|EST1D_RAT  T-LMGYPLSEGKLDQKTAKSLLWKSYPTLKISEKMIPVVAEKYFGGTDDPAKRKDLFQDL 418
SP|P23141|EST1_HUMAN MQLMSYPLSEGQLDQKTAMSLLWKSYPLVCIAKELIPEATEKYLGGTDDTVKKKDLFLDL 420
                       **.******:****** ******** : *::::** .:***:***** .*:**** **
SP|P16303|EST1D_RAT  VADVMFGVPSVMVSRSHRDAGAPTFMYEFEYRPSFVSAMRPKTVIGDHGDELFSVFGSPF 478
SP|P23141|EST1_HUMAN IADVMFGVPSVIVARNHRDAGAPTYMYEFQYRPSFSSDMKPKTVIGDHGDELFSVFGAPF 480
                     :**********:*:*.********:****:***** * *:*****************:**
SP|P16303|EST1D_RAT  LKDGASEEETNLSKMVMKYWANFARNGNPNGGGLPHWPEYDQKEGYLKIGASTQAAQRLK 538
SP|P23141|EST1_HUMAN LKEGASEEEIRLSKMVMKFWANFARNGNPNGEGLPHWPEYNQKEGYLQIGANTQAAQKLK 540
                     **:****** .*******:************ ********:******:***.*****:**
SP|P16303|EST1D_RAT  DKEVAFWSELRAKEAAEEPSHWKHVEL 565
SP|P23141|EST1_HUMAN DKEVAFWTNLFAKKAVEKPPQTEHIEL 567
                     *******::* **:*.*:* : :*:**
```

Figure S1: The primary sequence alignment of human and rat carboxylesterase

```
SP|P16303|EST1D_RAT  GGGESAKTLVGL 141,142,143,220,221,222,225,382,386,387,419,467,470
SP|P23141|EST1_HUMAN GGGESAKLVIGL 141,142,143,220,221,222,225,384,388,389,421,469,472
```

Figure S2: The key amino acids of the binding site sequence alignment of human and rat carboxylesterase

Results and discussion

All methods were validated and showed specificity, linearity (concentration range 1.25-250 µM, r >0.98), accuracy (relative error (RE) <15%), precision (relative standard deviation (RSD) < 15%), sensitivity (Lower Limit of Quantification (LLOQ) between 1.25-15 µM) and extraction efficiency (recovery > 84%). Table S1 shows linearity and sensitivity data and table S2 exhibit recovery, accuracy and precision data. The selectivity profile can be seen in table S3.

Table S1. Linearity and sensitivity data of esters compounds (**1-12**)

**Analytes**	**Range Concentration (µmol.L^{-1})**	**LLOQ (µmol.L^{-1})**	**Linear equation**	**r^2**
1	**10-250**	10	Y= 0.004023x – 0.08922	0.9801
2	**10-250**	10	Y=0.006257x + 0.09522	0.9863
3	**5-250**	5	Y=0.001801x + 0.1423	0.9842
4	**6.25-250**	6.25	Y=0.003256x – 0.009175	0.9939
5	**3.75-250**	3.75	Y=0.006454x – 0.01899	0.9944
6	**10-250**	10	Y=0.002317x – 0.008561	0.9969
7	**2.25-250**	2.25	Y=0.006978x + 0.03537	0.9920
8	**1.25-250**	1.25	Y=0.007064x + 0.06976	0.9949
9	**1.25-250**	1.25	Y=0.004028x – 0.005617	0.9933
10	**15-250**	15	Y=0.009080x – 0.04686	0.9898
11	**15-250**	15	Y=0.008738x – 0.01830	0.9935
12	**15-250**	15	Y=0.006613x – 0.0003978	0.9912

Table S2. Recovery, accuracy and precision data of esters compounds (**1-12**)

**Analytes**	**Nominal Concentration (μmol.L^{-1})**	***Recovery (%)***	***Intra-day***		***Inter-day***	
			Precision (RSD %)	Accuracy (RE%)	Precision (RSD %)	Accuracy (RE%)
**1**	**62.5**	98.6	3.50	1.40	2.96	3.95
	**125**	93.7	5.09	6.27	3.44	3.63
	**156.25**	96.7	0.59	3.29	2.26	5.69
**2**	**62.5**	90.4	7.82	9.54	6.80	7.83
	**125**	92.4	0.95	7.62	1.38	5.96
	**156.25**	95.7	0.74	4.34	1.03	2.93
**3**	**62.5**	88.4	3.09	11.6	2.90	9.54
	**125**	90.6	2.52	9.36	2.08	5.85
	**156.25**	92.5	0.24	7.48	1.74	5.23
**4**	**62.5**	94.5	1.06	5.45	1.43	6.78
	**125**	96.9	5.62	3.04	4.42	2.91
	**156.25**	95.3	3.88	4.70	3.44	2.46
**5**	**62.5**	91.5	4.51	8.46	2.51	5.23
	**125**	93.7	4.32	6.25	2.54	2.14
	**156.25**	95.7	2.58	4.31	3.35	8.21
**6**	**62.5**	90.7	1.22	9.29	0.98	3.74
	**125**	84.9	4.22	14.1	4.23	7.41
	**156.25**	91.1	1.14	8.90	3.02	8.11
**7**	**62.5**	92.5	2.01	4.13	1.21	7.65
	**125**	90.2	0.29	3.72	0.74	4.81
	**156.25**	92.4	4.05	3.94	3.83	1.93
**8**	**62.5**	104.1	2.01	4.13	1.21	4.34
	**125**	96.3	0.29	3.72	0.74	5.52
	**156.25**	96.0	4.05	3.94	3.83	3.11
**9**	**62.5**	96.4	8.16	3.56	9.43	2.96
	**125**	99.2	2.30	0.78	2.97	3.29
	**156.25**	109.9	2.78	9.90	4.65	4.21
**10**	**62.5**	94.0	2.60	6.02	3.20	5.94
	**125**	107.3	0.77	7.28	1.05	3.41
	**156.25**	107.6	1.20	7.63	1.68	3.98
**11**	**62.5**	88.4	2.60	11.6	2.51	8.14
	**125**	90.4	0.76	9.58	0.87	8.97
	**156.25**	88.0	1.20	12.0	1.39	5.64
**12**	**62.5**	104.7	2.59	4.67	2.14	6.34
	**125**	101.8	1.58	1.85	2.47	3.61
	**156.25**	109.1	0.39	9.11	0.94	6.47

Table S3. Selectivity data of esters compounds (**1-12**)

**Analytes**	**R_T analyte**	**R_T internal standard**	**R_T biological matrix**
1	8.7 min	15.2 min	1-3 min
2	10.7 min	15.1 min	1-3 min
3	13.0 min	15.1 min	1-3 min
4	15.3 min	14.2 min	1-3 min
5	14.0 min	15.7 min	1-3 min
6	14.5 min	14.9 min	1-3 min
7	12.6 min	14.6 min	1-3 min
8	10.9 min	11.6 min	1-3 min
9	7.7 min	11.7 min	1-3 min
10	11.9 min	14.2 min	1-3 min
11	14.7 min	14.3 min	1-3 min
12	14.7 min	14.3 min	1-3 min
